# Enactive Mind Autism: Autopoietic Enactivism as a Practical Framework for Autism Healthcare

**DOI:** 10.1159/psp/aelag010

**Published:** 2026-09-21

**Authors:** Johannes Cornelis van Huizen, Jan-Pieter Teunisse, Antje Orgassa, Wouter G. Staal, Derek W. Strijbos

**Affiliations:** a School of Social Studies, HAN University of Applied Sciences, Nijmegen, The Netherlands; b Research and Development, Dr. Leo Kannerhuis Centre for Autism, Arnhem, The Netherlands; c Department of Psychiatry, Radboud University Medical Centre, Nijmegen, The Netherlands; d Leiden Institute for Brain and Cognition (LIBC), Leiden University, Leiden, The Netherlands; e University Centre, Karakter Child and Adolescent Psychiatry, Nijmegen, The Netherlands; f Faculty of Philosophy, Theology, and Religious Studies, Radboud University, Nijmegen, The Netherlands; g Specialist Centre for Developmental Disorders, Dimence Group Mental Health Centre, Deventer, The Netherlands

**Keywords:** Autism, Complexity, Enactivism, Self-regulation, Sense-making, Speculative design

## Abstract

**Introduction:**

Autism healthcare has traditionally focused on individual biological and behavioral factors. In recent decades, there has been growing recognition that the outside world also plays an enabling if not constitutive role in the development of support needs. This broader view complicates healthcare delivery as the traditional person-centered focus ‘diffuses’ into the environment. An integrative approach, rooted in autopoietic enactivism, can provide guidance here – aimed at establishing stable, authentic, and health-promoting patterns of engagement between an autistic individual and their surroundings. Although the application of autopoietic enactivism has gained popularity among philosophers of psychiatry, its clinical consolidation remains limited.

**Methods:**

In addressing this gap, this paper presents a preliminary practical framework called enactive mind autism (EMA). EMA consists of five dimensions to explore the relevance and implications of an autopoietic-enactive approach in autism healthcare: aspects of (1) ecological, (2) historical, (3) tangible-embodied, (4) intersubjective, and (5) socio-cultural life.

**Conclusion:**

To conclude, this paper draws a connection with design research to imagine new methodological directions for clinical research and practice, which, from an autopoietic-enactive perspective, is encouraged to take a phenomenological, pragmatic, and material turn.

## Introduction

### Autism Healthcare Complexity

Clinically, autism is defined as a *neurodevelopmental disorder* characterized by “persistent deficits in social communication and social interaction,” as well as “restricted, repetitive patterns of behavior, interests, or activities” [1]. This definition foregrounds its biomedical origin and characterization, which typically focuses on the individual. In recent decades, a series of overlapping movements and approaches have sought to complement this view by framing support needs as situated in specific physical and socio-cultural contexts. Inspired by the social model approach to autism [2, 3], various complementary perspectives, such as the neurodiversity movement [4–6], critical autism studies and intersectionality [7, 8], as well as the autistic self-advocacy movement [9–11], build on the idea that autism research must move beyond individual pathology and engage with the ‘outside world.’ Approximately 1 in a 100 people is estimated to be autistic [12].

To adapt to these changing contours, autism healthcare must cultivate *contextual approaches* to support needs. This development is already underway, as can be seen, for example, in so-called neurodiversity-affirming healthcare, which emphasizes relational, holistic, strengths-based, and personalized approaches [13–17]. These approaches contrast with traditional individualist approaches – be it biomedical, behaviorist, or cognitivist in orientation – that place the locus of change within the autistic person. Applied Behavior Analysis [18, 19] and ‘mindreading’ trainings [20] are often cited as examples in this regard. By seriously considering the enabling if not constitutive role of the outside world, the idea is that contextual approaches can do better justice to the multifaceted reality of support needs.

At the same time, contextual approaches face their own challenges. Central to this study, contextual approaches considerably complicate the *delivery* of healthcare. A practical convenience of individualist approaches, namely, is that care may be confined to the person; with contextual approaches, the locus of care ‘diffuses’ from the individual into their surroundings. These surroundings are *complex* and *dynamic*, spanning various life domains and social networks [21–24], making it difficult for care providers to determine (in advance) which factors are causally relevant to a specific support need and how these factors may interact with one another [25]. This complexity also involves that care providers step outside of their traditional silos and connect with the practices of other formal and informal care partners [21], which is a persistent clinical challenge. Then, there is the inextricable question of normativity [26]: when should healthcare privilege reshaping the person’s environment, and when is it more appropriate to encourage change in the person’s own behavior by means of medication or behavioral interventions? In contextual approaches, moreover, biological and psychological components traditionally associated with individualist approaches may no longer receive sufficient attention. Although these components should indeed not be the exclusive focus of care, they remain relevant to the person’s support need.

### An *Integrative Approach:* Autopoietic Enactivism

An *integrative approach,* rooted in *autopoietic enactivism*, can help address these and other questions by enabling care providers to interpret and respond to support needs as emerging from the dynamic interplay between various (disrupting) factors^1^ [25, 27]. Autopoietic enactivism^2^ is a division in 4E cognition science that holds that cognition *emerges* through an organism’s situated and selective interaction with the world [28–33], drawing on insights from philosophical pragmatism, phenomenology, ecological psychology, biological and complex systems theory [34–36]. Among several interlocking concepts [36], two themes have been highlighted as entry points for clinical practice [37]: self-regulation and sense-making.

*Self-regulation* refers to the capacity of organisms to actively preserve themselves and maintain their identities by regulating their exchanges with the environment in order to fulfill their needs, particularly through moving around in the environment and changing it. Consider how one might avoid shopping in busy stores to prevent overwhelming sensations or cultivate relaxation by leisurely painting sessions. In autopoietic-enactive theory, this ability of organisms to actively govern or ‘regulate’ the terms of its interaction with the environment is referred to as *autonomy* [36]. The term is used to highlight the fact that the identities an organism preserves are not externally imposed, but rather emerge ‘from within,’ i.e., from the self-organizational activity of living beings themselves. Self-regulation happens at multiple identity levels [25, 38], related to – sometimes counteracting – basic needs such as bodily well-being and more socio-cultural concerns such as being liked and respected by the community.

Closely tied to the notions of self-regulation and autonomy is the concept of *sense-making* – the idea that living beings actively construct meaning from their needful interactions with the world. For example, the busy store and the leisurely painting sessions are meaningful in the autopoietic sense that they bear on the person’s well-being: the busy store raises anxiety and thus diminishes well-being; painting provides tranquility and thus enhances it. Sense-making, therefore, is an *affective activity* [25, 35, 39], involving the connection between the subjective significance of elements for a person’s autonomy, and how ‘good,’ ‘bad,’ or simply neutral, these elements are typically perceived. Naturally, it differs per organism what in their environment is meaningful [40, 41], and differences exist also within the ‘human spectrum.’ Not everyone is put off by busy stores, just like not everyone enjoys painting. Combined, sense-making and self-regulation undergird an organism’s ability to navigate an uncertain and at times unwelcome world, toward safety and flourishing and away from danger and ill-being.

Autism, and other forms of neurodivergence, can be seen as involving structurally *atypical forms of sense-making* [25, 38, 42]. For example, on average, an autistic person might be more easily overwhelmed by a crowded store than their neuro-average peer, just like, on average, they might also experience greater joy from esthetic patterns in art [38, 43]. These differences can be attributed to autistic embodiment: the particular ways “in which the biology, neurophysiology, affective, and sensorimotor structures and skills of people with autism differ from those of non-autistics” [38] – which is not so much about isolated bodily phenomena but about lived bodies in embodied environments [44]. Importantly, atypical forms of sense-making coincide with *atypical forms of self-regulation*. Spatial experiences may lead autistic individuals to navigate urban spaces according to fixed routes [45], while eye contact avoidance can be viewed as a strategy to regulate interpersonal engagement [38, 46]. Atypical self-regulation strategies can slip into health-diminishing behaviors – for instance, when it leads to over-rigidity or social withdrawal – but could also be harmless and instead empower the individual in authentic ways.

When dealing with complex and dynamic lifeworlds, the autopoietic-enactive approach brings with it two clinical advantages. First, it does not curb complexity but presupposes it, taking the client’s *interactional social-ecological system* as the central unit of analysis. The aim here is to negotiate stable, authentic, and health-promoting *patterns of engagement* between an embodied individual and their surroundings^3^. Second, in line with renewed appreciation of phenomenological inquiry among both cognitive researchers and neurodiversity advocates [47–51], the autopoietic-enactive account takes seriously the *lived experiences* of autistic individuals as an epistemic resource. Whereas cognitivist approaches tend to render experience epiphenomenal [36], autopoietic enactivism regards lived experience as informative of values, concerns, motivations, perceptions, and preferences that help orient the organism toward skillful engagement with its world – that is, to maintain their autonomy [39, 52]. Sense-making is indeed an inherently affective activity. In brief, the autopoietic-enactive approach not only broadens the clinical scope to encompass the person-environment system as a whole but also provides a means of navigating it by treating the client’s lived experiences, and their first-hand accounts of these experiences, as an epistemic anchor.

### *Paper Purpose:* ‘From Theory to Practice’

To date, autopoietic enactivism has been used to reinterpret mental healthcare in a broader sense [25, 53–57], as well as a range of specific psychiatric conditions such as schizophrenia [58, 59], depression [60], anxiety disorders [61], and, most extensively, autism [38, 42, 52, 62], which also forms the focus of this paper. Much of this work, however, remains theoretical. Autopoietic enactivism has been harnessed primarily by philosophers of psychiatry to reconceptualize support needs in ways that underline their contextual and interactional nature. In mainstream clinical practice, the relevance and implications of an autopoietic-enactive approach is still underexamined. Care providers might find it difficult to engage with autopoietic enactivism as a healthcare concept, or assess what benefits it could bring them. This ‘gap’ also concerns clinical research: without proper clinical consolidation, it remains difficult to determine a unified approach to scientific experimentation, validation, and progress. In sum, while autopoietic enactivism is promising in theory, its application in practice deserves more attention.

To this end, this paper presents a preliminary practical framework called *Enactive Mind Autism (EMA)*. The framework consists of five dimensions: (1) *ecological*, (2) *historical,* (3) *tangible-embodied*, (4) *intersubjective,* and (5) *socio-cultural life* (shown in Fig. 1). EMA does not so much describe a single autopoietic-enactive method, but as mentioned, explores the relevance and implications of an autopoietic-enactive approach to the clinical domain. Rather than prioritizing theoretical coherence, we have thus arrived at these dimensions practically and iteratively: from the perspective of the care provider, what autopoietic-enactive considerations might they encounter in day-to-day clinical practice? In the Discussion section, we briefly discuss opportunities to extend and refine the framework in this regard.

**Fig. 1.**
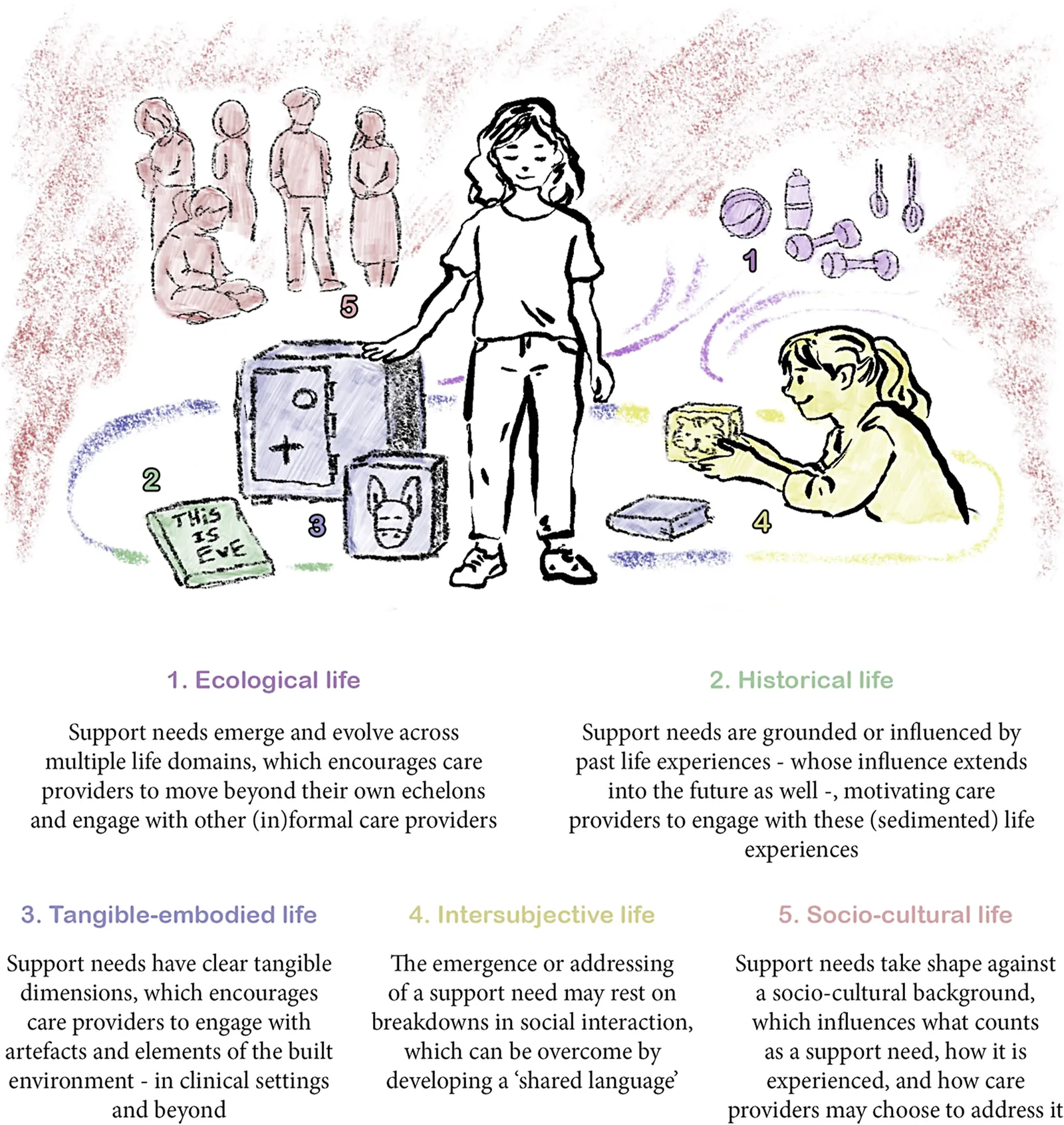
Five dimensions of enactive mind autism (EMA).

In what follows, EMA’s five dimensions are presented consistently using four parts. First, (A) we draw on autopoietic-enactive and related theories to introduce and substantiate each dimension. Where relevant, (B) we highlight existing healthcare practices that connect to the dimension in question^4^. The purpose of this is twofold: (1) to show that some of the underlying ideas are not entirely new and that care providers already work in ‘eclectic’ and ‘holistic’ ways that could be described as autopoietic-enactive [25]; and, (2), by accommodating these healthcare practices in a unified framework, it fosters exactly the kind of integration that the psychiatric field is moving toward [63, 64]. After this, (C) we illustrate each dimension using a vignette: *The Story of Eve*^5^, based on a real-world case study with a 10-year old autistic girl. Finally, (D) we present a fictional yet realistic ‘speculative design’ to explore and imagine what an autopoietic-enactive approach might have entailed in this case. In design research, speculative design is used to challenge contemporary practices and envision possible futures through critical, provocative, and forward-looking artefacts and applications [65, 66]. The idea is that engaging with speculative design compels us to move beyond the theoretical realm and into the realm of imaginative clinical practice. We begin this paper with the vignette.

### The Story of Eve: ‘Keeping the Tiger Small’

Eve is 10 years old and lives with her parents and younger sister. Until 2 years ago, things at home were difficult: there were frequent arguments, and playing with her sister often ended up in conflict. As part of her treatment at a Dutch healthcare organization, she also received support at school through *Behandeling in de Klas – Treatment in the Classroom (BINK)*, where a counselor observed in class and offered guidance. Eve had previously attended a care farm, but since taking up athletics twice a week, she feels she no longer needs this.

A turning point came when Eve and her family entered family therapy. On advice of the healthcare organization, the family spent a week in the countryside, where Eve and her family learned strategies for managing emotions and conflicts more effectively. Since then, there have been clearer rules at home – for example, ‘stop’ during play now really means stop. When conflicts arise, a one-on-one conversation works best, rather than everyone becoming involved. Breaking agreements has consequences, such as losing screen time – and this applies to the whole family, including the parents. After the admission, the family members also took part in follow-up ‘reset’ sessions to help prevent slipping back into old patterns. Since their week in the countryside, the family has been able to undertake more activities together, such as going on family holidays.

One key element of the family therapy was the ‘tiger.’ Each family member received a block depicting a tiger, which represented anger. The aim was to ‘keep the tiger small,’ meaning to take a step back and do something else when feeling frustrated. At home, Eve keeps her tiger small by going to her room or spending some time on her tablet.

## Methods

### Aspects of Ecological Life

To begin, what does it mean to take the client’s interactional social-ecological system as the central unit of analysis? First, it means to recognize the *scope* of said system: support needs emerge and evolve in interaction across different life domains and may be addressed through different types of (in)formal care in accordance with these domains. Existing cross-sectoral care approaches already tap into this by seeking to improve coordination across healthcare, educational, home, and policy contexts [67–70]. From an autopoietic-enactive perspective, what gets emphasized here is that these facets of care relate to each other in holistic ways [25, 27]: they are dynamically intertwined to form a bigger whole without a clear hierarchical structure that allows for reduction. Changes in one dimension can certainly influence changes in another, but the causal relations here are often non-linear and transformative. Changes in local processes can affect the emergent whole, which can reciprocally transform its local constituents. Through time, patterns of dynamic causality unfold that do not allow for simple reconstruction in terms of stable root causes and their effects.

One implication of this is that healthcare interventions cannot be used for *‘reverse engineering’* – addressing one dimension without considering the disrupting factors in other dimensions – nor that targeting the ‘original factor,’ which is assumed to have caused the support need, guarantees an immediate or comprehensive solution of the problem [25, 71]. From a pragmatic healthcare perspective, mapping the broad scope of a person’s interactional system – as well as how support needs emerge and are addressed in it – is not a one-off assessment but a time-extensive activity *in and of itself* that involves multiple life domains and collaboration with (in)formal stakeholders.

#### Dynamic Care Poster

In the story of Eve, care was provided across multiple settings and in diverse forms. Formally, she received guidance at school, within a healthcare organization specializing in autism, and, until recently, at a care farm. Informally, Eve’s personal development was shaped at home through interactions with her parents and a sibling, as well as in her athletics class. This multiplicity of contexts and relationships makes it difficult to pinpoint where the conflicts at home originated. It also complicates the task of determining a suitable care plan: which types of care, in which settings and with whom, would be most appropriate for addressing these conflicts? And, importantly, how does intervention in one setting affect the others?

For the dimension of ‘ecological life,’ we envisioned the fictional *Dynamic Care Poster* (shown in Fig. 2). Together with her care providers, including her parents, Eve maps the various settings in which she undergoes care and self-development. This also includes her athletics class, which, even though it is no form of formal care, plays an important role in her life. Since attending athletics classes twice a week, Eve no longer felt the need to stay at the care farm. At the same time, it is recognized that this may change as Eve continues to grow. To keep track of these developments, the poster also includes a timeline. Each month, Eve gets together with her care providers to discuss what they have observed across life domains. Significant changes are recorded and reflected upon, including how insights from one setting relate to and influence others.

**Fig. 2.**
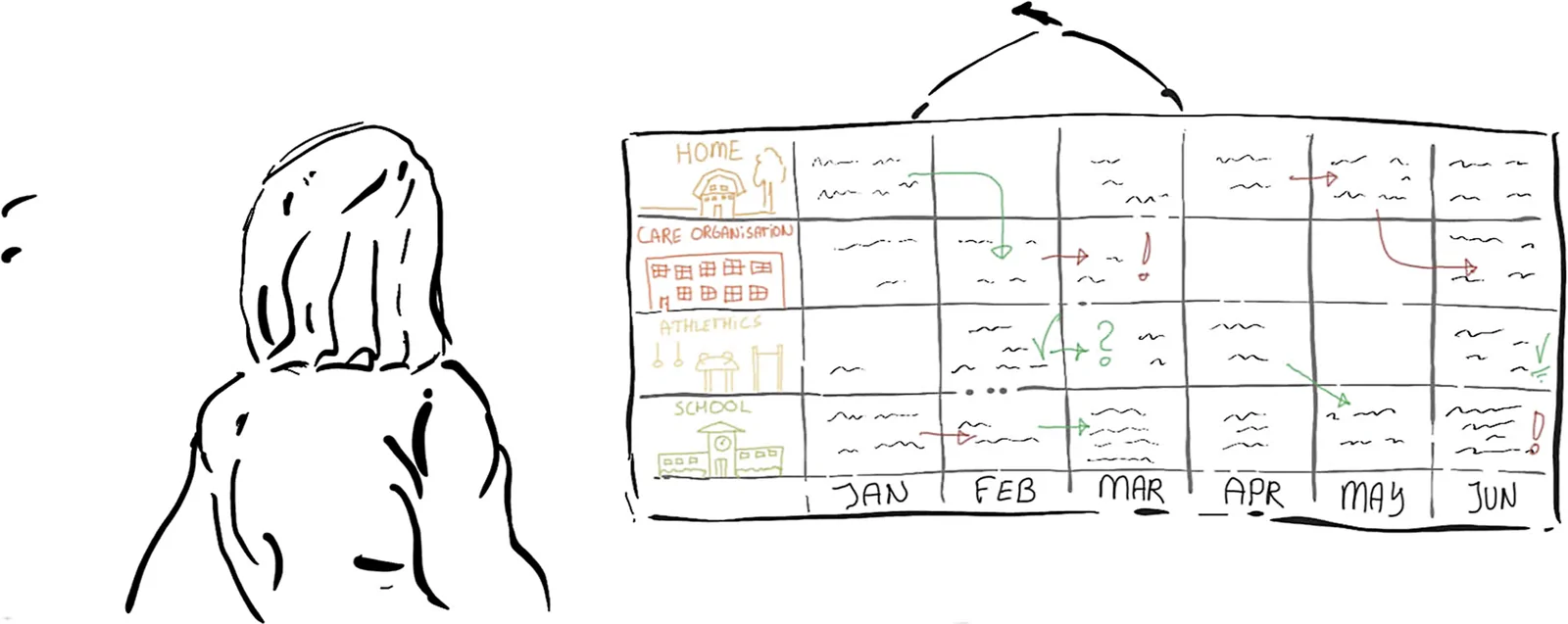
The dynamic care poster helps Eve and her family obtain a holistic overview of relevant life domains and how support needs evolve in it – an ‘anchor point’ for shared sense-making.

### Aspects of Historical Life

The complexity of the social-ecological interactional system also extends in *time*. The question of why and how we experience interactions in the world and how this influences our actions pertains to more than just a ‘screenshot.’ Complex systems are essentially historical, meaning that their unfolding behavior is shaped by non-linear, path-dependent sequences of past interactions. Past life experiences nurture how the present is perceived, experienced, and they open or constrain possible futures.

Van Huizen et al. [72, 73] provide simple yet illuminating examples. In their study on supportive technology, the researchers paid particular attention to the young adults’ embodied experiences with artefacts and the built environment. One participant explained that, of the two fidget spinners she used to regulate sensory stimulation, her favorite was the one she had bought at a theme park with her friends. Another participant described his laptop as ‘an extension of himself,’ noting that, because of his slim physique, it had made his time in school easier by allowing him to access study materials online rather than carrying heavy textbooks. The meaning of these respective aids to the participants cannot be separated from their use contexts [74, 75]. For the fidget spinner, the cherished memory attached to it even outweighed the significance of its technical features: the participant explained that the unused fidget spinner had, in principle, more potential because of its double fidgeting axes. Still, she preferred the theme park one.

In these examples, it is helpful that the participants were able to link their experiences to a specific event and episode, respectively. However, through habit formation, experiences may become *sedimented*: influencing how we perceive, experience, and act in the presence even when they no longer evoke a specific memory [76–78]. Furthermore, major events – painful and traumatic events [76, 79], for example – may drastically and enduringly change how a person behaves in relation to their surroundings, even when the event itself has settled into the background of consciousness. In these cases, events and episodes appear no longer as discrete occurrences but have sedimented into an experiential background against which new events are made sense of. In this regard, De Haan [25] speaks of an *existential* dimension that highlights the ways in which we relate to ourselves and our own existence, that has clinical relevance: of “[how] our expectations can influence the effect of medication, our fear of future harm influences our behavior, our shame affects our posture as well as our social interactions.” Psychotherapy, such as mindfulness therapy and acceptance and commitment therapy, can help address this existential dimension [25].

#### ‘This is Eve’ Book

In Eve’s story, it can be assumed that past life experiences play a role in how she perceives the present, even if they cannot be traced back to a single event. Conflicts at home may have left lasting impressions, yet it remains unclear how Eve understands her own role in them. How does she look back on these episodes of her life? Are there experiences that remain unsettled?

For the dimension of ‘historical life,’ we envisioned the fictional *‘This is Eve’ Book* (shown in Fig. 3). Using an instant camera, Eve is invited to photograph artefacts, spaces, people, and other elements in her environment that evoke certain feelings – both positive and negative. The photography exercise can help resurface said sedimented experiences, and the book itself can serve as a springboard for dialog, providing a platform to (collaboratively) explore how past life experiences continue to play into the present.

**Fig. 3.**
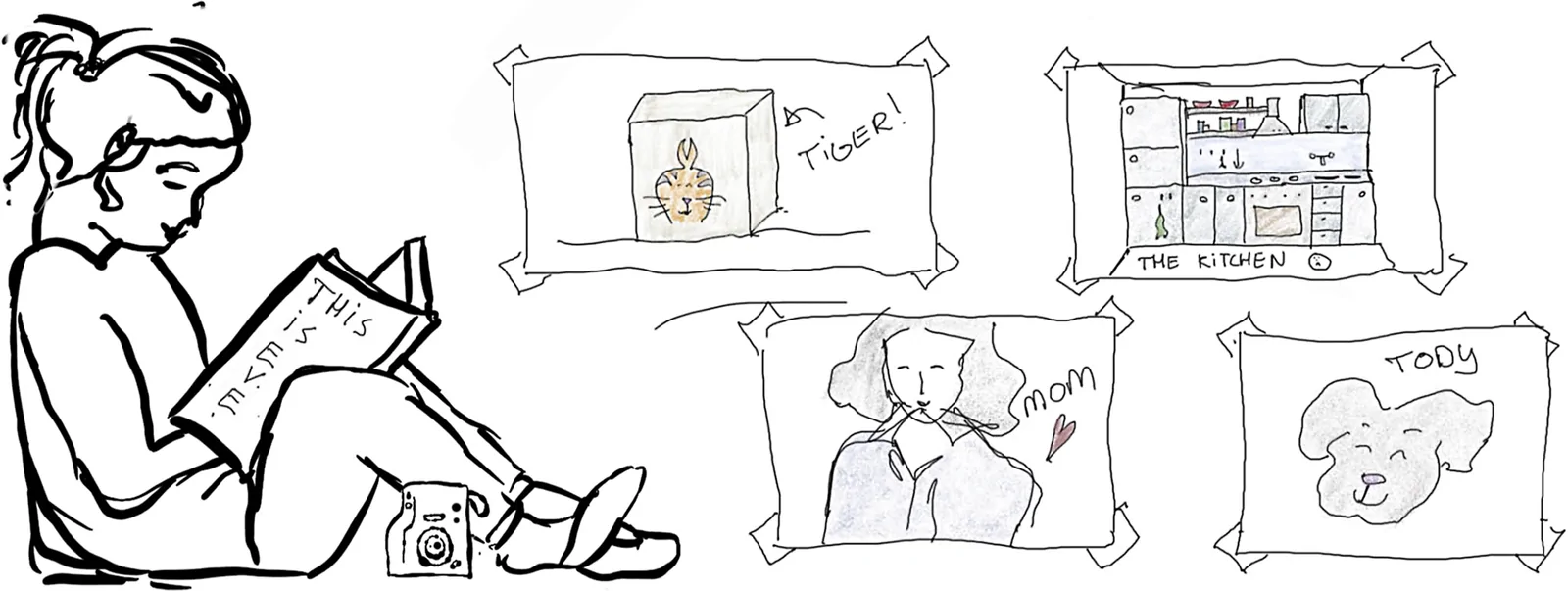
The ‘This Is Eve’ Book offers a springboard to Eve and relevant others to discuss meaningful elements in Eve’s life, exploring how past experiences continue to influence present experiences.

### Aspects of Tangible-Embodied Life

As mentioned, an autopoietic-enactive approach takes the interactional system of the organism or person as the central unit of analysis. These interactions unfold in space and over time. Zooming in, a person’s interactional system has clear tangible dimensions; the lifeworld is *embodied*. In the story of Eve, for example, it is worth considering which *artifacts* and elements of the *built environment* played a role in how she experienced school, home, and the care farm, and how these experiences have influenced the development of support needs.

In the literature, it is suggested that autistic individuals may benefit from constructing an ‘eco-niche,’ referring, in part, to tailored material conditions that afford individuals to move comfortably in and across physical spaces [25, 42, 52, 80]. Nešić et al. [81] exemplify what such an eco-niche could look like by referring to the medieval monastery, which they argue may have offered a safe space to autistic individuals due to its predictable and low-intensity sensory environment. In current research, tangible considerations relate to modifying the sensory conditions of the clinical setting [82], as well as using physical objects [83, 84] and even animals [85, 86] to mediate and support the therapeutic exchange.

Noteworthy is that, in design research, there is branch that engages with phenomenology more thoroughly – in interaction design in general [87–89], and in the context of autism more specifically [45, 72, 90–92]. The objective here is to create artefacts that integrate into, help sustain or transform the person’s lifeworld so that they are better able to cope with and control their surroundings. For many autistic individuals, clever use of artefacts and adaptations in the built environment can contribute to the regulation of everyday challenges. Examples include the installation of carpet floors over noisy laminated floors [93], and the use of fidget spinners and noise-canceling headphones to enhance sensory regulation [94, 95].

Upon reflection, it is also under this dimension that care providers can consider *pharmacological* interventions – not as isolated interventions in the physical body, but in the interaction between a person’s lived body and their social and physical environment [57]. At stated in the introduction, preferences for interaction are partially yet undeniably rooted in biological and physiological organization [96–98]. Consider the use of MDMA, which leads to an increase in oxytocin levels and can help improve feelings of connectedness in dyadic interactions [99, 100]. MDMA, like other pharmacological agents, is a tangible environmental tweak – a *technology* of some sort [101], much like fidget spinners and noise-canceling headphones – that can be used to positively influence interaction.

#### Screen Time Safe

In the story of Eve, slipping back into old patterns remained a real concern. While in the countryside, the family learned new strategies for managing emotions and conflicts, supported by clear rules. The concern here is that these strategies are tied to the specific context where they were taught, raising the question of whether they could be successfully carried over and applied in the family’s home environment [102, 103].

Recreating the setting at home would make little sense, but it is conceivable that the family could at least experiment creatively with tweaking their own physical surroundings to facilitate the transfer of newly learned skills. For the dimension of ‘tangible-embodied life,’ we, therefore, envisioned a fictional *screen time safe* (shown in Fig. 4): a tangible artefact that helps the family introduce and sustain the new rules. Family members who break the new rules must place their phone, tablet, or laptop in the safe, which opens automatically after 2 h. Although the safe does not eliminate the need for mutual policing, the rules are more transparent, ‘formalized,’ and feel more equitable. After all, also her parents must place their screen into the safe when they break the rules. Eve feels less resentment toward her parents when she loses her screen time: it is the safe that is unyielding, and the timer makes it clear how long she still needs to wait to get her screen back. In addition to the screen time safe, Eve is given a small dose of risperidone (0.25 mg) to support her in regulating her behavioral and emotional responses.

**Fig. 4.**
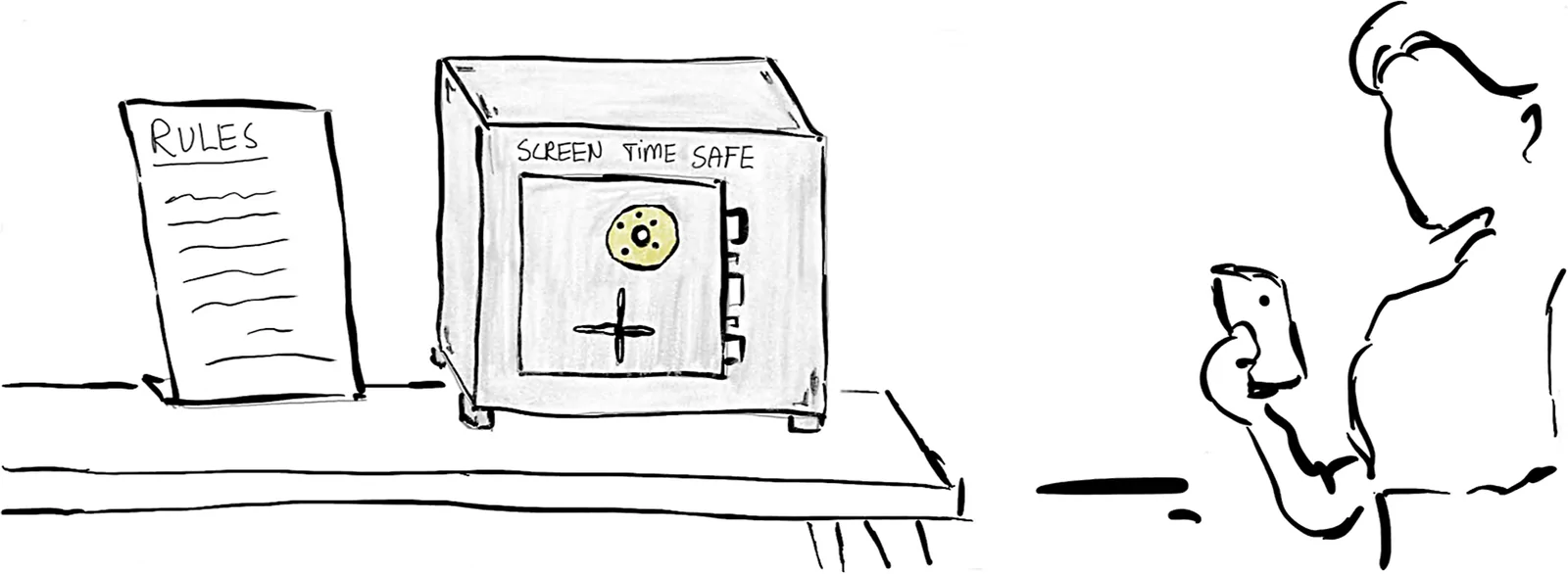
The screen time safe helps the family adopt new rules, introducing an ‘exterior authority.’

### Aspects of Intersubjective Life

Other people play a major role in our lives. They may assist in self-regulation and sense-making. In the clinical context, care providers and trusted others can function as co-regulators, helping individuals to structure their interaction with themselves and their surroundings so as to better fulfill their current needs. They may also support the process of meaning-making by exploring means of expression and facilitating reflection on oneself, their social relationships, and the world at large. This can help a person experience and (re)view their support needs from a different angle, while care providers gain insight into the person’s perceptions and experiences. Through interaction, other people may thusly contribute to developing a sense of agency, autonomy, and self [104–106].

Such shared sense-making, or *participatory sense-making* [107], however, relies on the establishment of intersubjective engagement. To a critical extent, interaction partners need to ‘speak the same language.’ This may be challenging in neuromixed settings due to “alterations of embodiment, temporality, and intersubjectivity” [108]. As a result, autistic and non-autistic individuals show different preferences for *interaction styles* [38, 109–111]. A well-known example is that autistic people may prefer not to look others in the eyes [46], or take longer pauses in conversations [112]. Also echolalia can coincide with autism, which may be viewed as a strategy to affirm oneself in the interaction [38, 113].

In the literature, various suggestions are made for achieving resonance in interaction, which, from a contextual perspective, can be viewed as a shared responsibility [114]. For verbal communication, it is suggested that the care provider matches the child’s volume and prosody [115], and their preferred level of detail when discussing self-chosen topics [116], among others. For non-verbal interaction, it is suggested to establish intersubjective engagement through bodily and motor synchrony, for example, through dance/movement therapy [104, 108, 117], or rhythmic and musical approaches [82, 118, 119]. The underlying idea here is that, for both verbal and non-verbal communication, interaction partners become attentive to each other’s social cues that enable or support conversations and other forms of empathic engagement – creating a ‘we-space’ [44, 120].

#### Animal Blocks

In the story of Eve, it is mentioned that the family often experienced conflicts and arguments. The parents describe how, when Eve gets angry, she makes herself appear large – ‘like a tiger.’ During the family’s stay in the countryside, this metaphor was incorporated into therapy: each family member received a wooden block depicting a tiger, symbolizing anger. The aim is to ‘keep the tiger small,’ meaning to prevent becoming overwhelmed and fretful. Eve keeps her tiger small by retreating to her room or spending some time on her tablet.

For the dimension of ‘intersubjective life,’ we envisioned fictional *animal blocks* (shown in Fig. 5). In addition to the tiger, other blocks depicting a donkey, a sloth, and a dog were added, representing sadness, fatigue, and happiness, respectively. Eve and her family can use the blocks to express their state of mind when they cannot, or do not wish to, put it in words. The blocks may also serve as prompts for conversations about each other’s feelings. Effective about this idea is that it builds on an existing metaphor – one that was *shared* and *understood* by all family members – while the introduction of other animals expands it in creative ways.

**Fig. 5.**
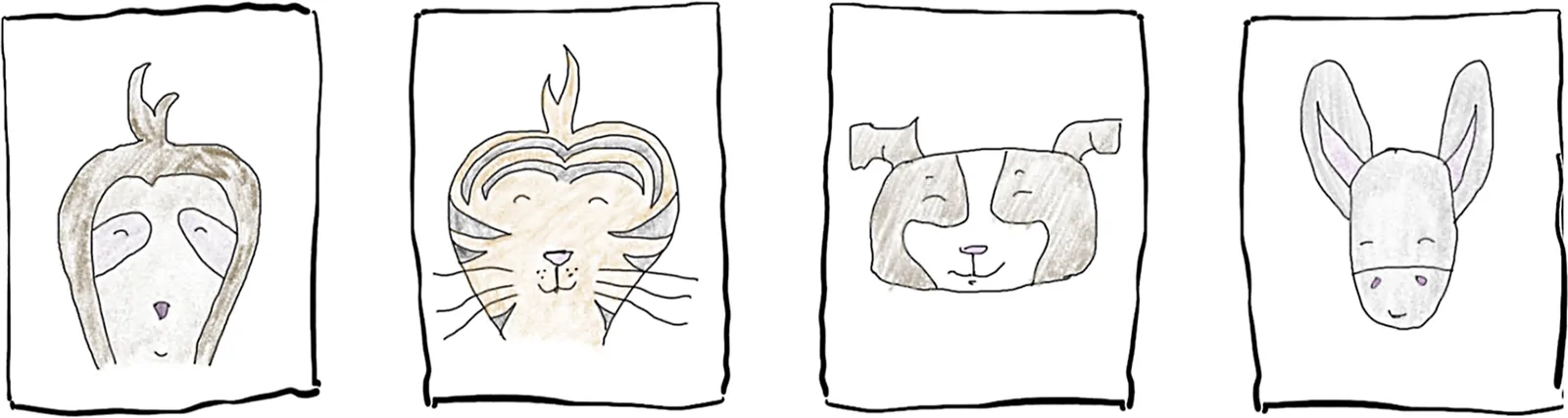
The animal blocks offer the family members a shared channel for communication, building on a pre-existing metaphor – the tiger symbolizing ‘anger’.

### Aspects of Socio-Cultural Life

Finally, it is essential to realize that a person’s interactional system does not exist in a socio-cultural vacuum. The socio-cultural normative landscape in which we live facilitates but also constrains the range of possibilities we experience for regulating our interactions with the environment. Norms and underlying values structure interaction patterns that we are open to and close off others.

This dimension may be introduced using an example from the work of Wonink [personal communication February 8, 2026, 59], part of the overarching study by Van Huizen et al. [72] on supportive technology. In this study, a participant describes how she wants to use a fidget cube to suppress sensory overstimulation, but she also worries that it might make her seem ‘odd’ since it is not something people typically do. She wonders why it is considered normal to play with a sheet of paper or a lock of hair, but purchasing a fidget cube is considered strange, even though it serves the same purpose. In this example, why is the participant concerned about how other people view the fidget cube, potentially even wanting to hide it? One answer could be that it reflects *internalized stigma* [121–123]; the idea that autistic people conceal and camouflage their characteristics because they come to believe and adopt the negative stereotypes and prejudices that society holds about their community.

Internalized stigma, or *self-stigma*, illustrates how self-regulation unfolds and is experienced against a socio-cultural background. In the case of the fidget cube, the ‘fidgeting’ responds to a need for stimming; the ‘cube’ part reveals a concern to how far one may go in addressing that need while remaining within socially acceptable bounds. Social norms and expectations can help individuals organize society efficiently by determining a shared but often sedimented understanding of social roles, rules, and conventions for interaction [124, 125], but, by doing so, also reinforce standards of ‘normal behavior.’ Normality, however, is relative, and diverging from the average should not by necessity imply *impaired functionality*. Armstrong [126] provides the example of a Congolese tribesman, presumably autistic, who was not stigmatized but rather praised for his eye for detail and excellent weaving skills. Normality, and, in the context of healthcare, concepts such as ‘illness,’ ‘disability,’ or ‘disorder’ are not fixed concepts [127, 128].

In recent decades, definitions of autism and other forms of neurodivergence have been debated and (re)negotiated, driven by internal psychiatric forces and external forces in the form of civic activism [129–132]. The social model approach to autism, as well as the movements and frameworks that followed, have suggested an alternative socio-cultural horizon in which ‘fitting in’ is no longer the primary objective of healthcare. Instead, autistic individuals may take pride in their autism and rather seek to bridge the gap between themselves and their neuro-average surroundings through authentic patterns of engagement. Against this background, would the participant still have felt the need to hide their fidgeting?

#### The EMA Discussion Group

For the dimension of ‘socio-cultural life,’ we envisioned the fictional *EMA discussion group*: a platform that enables care providers to reflect on normative assumptions in healthcare practices and attitudes (shown in Fig. 6) – a platform to disrupt the ‘flow of tradition,’ which Gallager [77] describes as “a socialized practical habit.” In Eve’s story, it is worth considering why the care provider accepted the tiger metaphor as a valid means of communication. Also why, as part of this very study, Eve and her family were invited to tell their story from the perspective of their own lived experiences [9, 47, 133], in contrast to being rigidly interviewed based on a predetermined theory or framework – ‘fishblowled,’ that is [134, 135]. Questions concerning normativity are discussed in scientific and philosophical fora, but they are just as relevant for the care practice from which they emerge [84]. The EMA discussion group allows for such reflection-on-action^6^ [136]. It invites reflection on the normative assumptions that structure the socio-cultural background against which patterns of engagement are negotiated in healthcare practice.

**Fig. 6.**
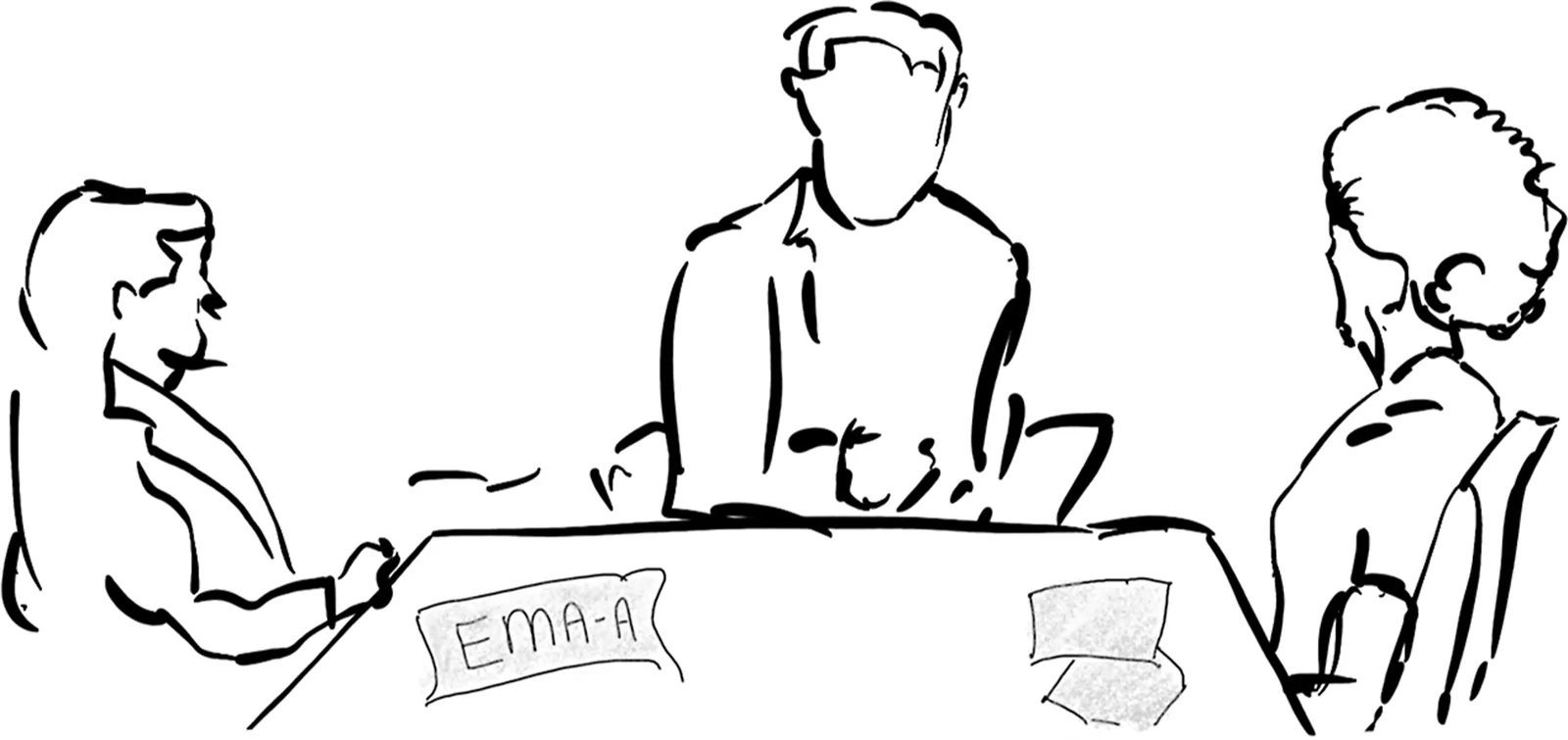
The EMA discussion group enables care providers and other stakeholders to discuss and explore the normative assumptions underlying their care practices.

## Discussion

### Summary

As an integrative framework, the autopoietic-enactive approach treats the interactional social-ecological system of the autistic person as the central unit of analysis. Under this approach, the objective of healthcare is to establish stable, authentic, and health-promoting patterns of engagement between an individual and their embodied environment – embracing rather than curbing complexity. At the same time, current autopoietic-enactive approaches are still in search of clinical consolidation, making it difficult for care providers to engage with it. To address this need, this paper proposed the *Enactive Mind Autism framework* – not a single autopoietic-enactive method, but a practical framework to explore the relevance and implications of an autopoietic-enactive approach to the clinical domain.

EMA consists of five dimensions. In summary, (1) *ecological life* stresses that support needs emerge and evolve across multiple life domains. Cross-sectoral approaches tap into this by encouraging care providers and relevant others to collaborate beyond their own echelons; (2) *historical life* places support needs in a temporal context, encouraging care providers to engage with (sedimented) past life experiences and how they influence current interaction patterns; (3) *tangible-embodied life* highlights the material constituents of everyday life, noting that patterns of engagement depend, in part, on physically cleverly constructed ‘eco-niches’ intrinsically related to specifics of individual embodiment. Here, phenomenology-informed design approaches and projects can offer a source of inspiration; (4) *intersubjective life*, then, zooms in on the social dimension of these eco-niches, pointing out that breakdowns in social interaction may be overcome by finding a ‘shared language’ – achievable through verbal means and non-verbal means such as bodily tuning and motor synchrony; finally, *(5) socio-cultural life* relates to how healthcare *should* be organized, recognizing that support needs are shaped against a socio-cultural background. Patterns of engagement are ‘negotiated’ against this background. Care providers are motivated to reflect on the normative assumptions that underly their healthcare practices.

### *EMA:* An Enacted Framework

How EMA should be understood and used in practice comes with some conditions. First, while for usability reasons it is tempting to consider each dimension separately, it is essential not to lose sight of the ‘bigger picture.’ EMA’s dimensions are indissolubly linked. Consider Eve’s *Dynamic Care Poster*, designed to explore how her well-being may be supported across life domains (*ecological life*). It is also a tangible object, made of paper and pen, hanging in the family’s residence (*tangible-embodied life*). It provides value by offering shared vocabulary for care providers to discuss Eve’s support needs (*intersubjective life*), and helps determine who should be involved in addressing them (*socio-cultural life*). The value of distinction lies in the opportunity to identify potentially relevant entry points for intervention in a complex and dynamic support need. However, as interdependent aspects of a person’s interactional social-ecological system, these dimensions presuppose one another. In fact, part of the clinical utility of these dimensions may lie in the opportunity they offer to explore their reciprocal influence in an ongoing process of participatory sense-making between care providers and their clients. The five dimensions we have identified provide a starting point for further research and development. As mentioned, they are inspired by care providers’ insights into relevant aspects of support needs. They may prove more difficult to disentangle, both conceptually and empirically, under closer theoretical-philosophical and empirical scrutiny.

Second, as foreshadowed in the introduction, EMA likely does not cover *all* relevant dimensions. Indeed, it is a preliminary framework. In assessing and addressing a support need, one might, for example, also consider a religious or *spiritual* dimension. Also, what of lived bodily sensations? For practical purposes, *somatic sensations* – through allostatic feedback loops [137, 138] – might be considered a distinct dimension. In this regard, a potential avenue for future work could be narrative research into turning point experiences among autistic individuals: what other distinct dimensions of a person’s interactional social-ecological system can be identified that play a role in the emergence or development of a support need? While these turning points may be difficult to trace due to the complex, non-linear nature of said social-ecological systems, transdisciplinary research – through co-design, open dialog, and other methodological means – can bring together a multitude of perspectives and initiate a process of shared sense-making.

Third, implied by the former two conditions, EMA insists on enactment. It is not an explanatory theory for autistic cognition, but a practical guide to engage with (autistic) life. Also, life is *lived*, which sounds banal but is essential. Life domains are crossed (*ecological life*); artefacts are used and built environments are inhabited (*tangible-embodied life*); relevant and intimate others are engaged with (*intersubjective life*); past life experiences are sculpted through events and episodes (*historical life*); and socio-cultural norms and values settle in practice (*socio-cultural life*). Meaning emerges in interaction – a core principle of autopoietic enactivism and 4E cognition science more broadly. Consider again the *Dynamic Care Poster*, which seems sound in theory but could fail in practice. It might not work as intended, trigger stress and conflicts among its spectators, or simply be too bulky. It needs to be experienced in real-time and real-space to be effectively evaluated – in the *here and now*, where all ‘visible’ and ‘invisible’ dimensions of lived experience converge. EMA-inspired interventions require continuous probing and tinkering; their practical value is established by the relevant stakeholders in concrete clinical situations through a process of iterative evaluation and adjustment. Formulated more strongly, without enactment the framework would contradict the enactive epistemology on which it is built: without experienc*ing*, sense-mak*ing*, self-regulat*ing* – that is, without transformative *action* – it cannot engender the integrative *know-how* required to understand and address a person’s support need. Indeed, EMA is intended to structure, guide, and substantiate pragmatic action; not to produce an exhaustive (causal) representation of all relevant (disrupting) factors.

### *Enactive Methodology:* Drawing on Design Research

While a comprehensive methodological discussion lies beyond the scope of this paper, we conclude it with the critical observation that the clinical domain currently seems ill-equipped to conduct research that engages with the *‘here and now.’* Largely resting on traditional evidence-based paradigms, clinical research values linearity, standardization, and theoretical solidification. In contrast, an autopoietic-enactive approach values an iterative, explorative, pragmatic addressing of support needs within phenomenologically rich, idiosyncratic real-world personal contexts. Another tension concerns material engagement, which is rarely foregrounded in clinical discourse but is a central theme in autopoietic enactivism [139]. It is difficult to imagine a care plan that does not require or entail a physical configuration of some sort in a person’s interactional system. Still, attention to the everyday physical spaces and artefacts *through which* autistic individuals give concrete shape to their lives remains more a byproduct of care rather than a starting point. In brief, if clinical consolidation of the autopoietic-enactive approach is to be taken seriously, it must begin with a reimagining of clinical research heuristics regarding goal-setting, monitoring, and evaluation. Indeed, if the aim of the autopoietic-enactive approach is to ‘establish stable, authentic, and health-promoting patterns of engagement,’ how can clinical research define, study, support, and assess such processes in a scientifically robust manner?

To imagine an ‘enactive methodology,’ a somewhat unexpected yet illustrative comparison may be drawn with *design research* – an academic field that seeks to support and improve the systematic process by which researchers collect and analyze data about end user’s experiences and their surroundings to inform the development of artefacts. Design can be construed as an enactive activity *par excellence*, bolstered by their shared grounding in pragmatism: ‘knowing as doing,’ emphasizing situatedness and interactivity [35, 140, 141]. Unlike in rigid clinical frameworks, however, design research embraces its ‘fuzzy’ front end [142–145], acknowledging that the understanding and addressing of a challenge evolves in reciprocal relationship.

Much like the clinical domain seems increasingly compelled to do, design research has a substantial history of embracing complexity. As such, it also has had a head start developing epistemic and methodological constructs, resources, and discussions to engage with this complexity [146, 147]. For example, *framing and reframing* describes how problems are always viewed provisionally; redefined based on new insights [136, 144, 148, 149]. *Context-mapping techniques* provide creative means to bring the person’s lifeworld into the research space, for example using photography [143, 150–152]. *Prototyping* – construction, deployment, and assessment of prototypes – highlights in-situ knowledge production [89, 90, 153], while *co-design* supports what could be described as participatory sense-making between the researcher, end user, and other stakeholders [134, 145, 154, 155]. Also *speculative design*, as applied in the present study, may be viewed as an enactive method: bringing forth, or enacting, concrete possible futures based on contextualized scenarios and embodied interactions. While speculative design may have steered our fictional examples for Eve’s case toward tangible artefacts, the underlying valuation of creativity, situatedness, and interactivity – of ‘designerly thinking,’ that is – could also be applied to solutions to which care professionals may be more familiar, e.g., imagining new routes to school (*ecological life*) or weekly routines based on leisure activities (*historical life*), using pets to engage emphatically (*intersubjective life*), or setting up training modules to curb social discrimination in educational and professional environments (*socio-cultural life*). To conclude with an open question: what methodological insights could the clinical domain gain from design research if it were to consider healthcare interventions essentially as participatory human-centered *design processes*?

## Acknowledgments

We are incredibly grateful to Eve and her family for their participation. We also thank all consortium partners for their continuous involvement – for allowing us to work in ‘enactive’ ways ourselves. While writing this paper, ChatGPT (version GPT-5o) was used for editing purposes.

## Statement of Ethics

This study is part of the ‘*Enactive Mind Autisme: van Denkwijze naar werkwijze’* project, which was reviewed by the Medical Ethics Review Committee (METC) Oost-Nederland (dossier number METC Oost-Nederland: 2022-15727), following which it received an exemption from the Medical Research Involving Human Subjects Act (WMO). This sub-study, involving the collection of client narratives (including the presented case study of Eve and her family), was reviewed and approved by the Internal Testing Committee of Karakter Center for Child and Adolescent Psychiatry (with study number 215-22) and subsequently approved by the Executive Board of Karakter. Written informed consent was obtained from all participants involved in this study. For all participating parents and children (if applicable), written informed consent was obtained from both parents (or their legal guardians) as well as from the child. All participants were informed that the collected data may be used for academic and scientific purposes in an anonymized form.

## Conflict of Interest Statement

The authors have no conflicts of interest to declare.

## Funding Sources

This research was supported by the Dutch Taskforce for Applied Research SIA [RAAK.PRO.04.073]. The funder had no role in the design, data collection, data analysis, and reporting of this study.

## Author Contributions

Johannes Cornelis van Huizen: writing – original draft, conceptualization, methodology, investigation, data curation, and visualization. Jan-Pieter Teunisse: writing – review and editing, conceptualization, methodology, investigation, resources, data curation, supervision, and funding acquisition. Antje Orgassa: writing – review and editing, conceptualization, methodology, investigation, resources, data curation, supervision, project administration, and funding acquisition. Wouter G. Staal and Derek. W. Strijbos: writing – review and editing, conceptualization, methodology, investigation, resources, supervision, and funding acquisition.

## Data Availability

This study builds on a real-world case study involving Eve and her family, the data for which was collected using a semi-structured interview that was audio-recorded and transcribed. For privacy reasons, neither the audio recording nor the transcript is publicly available. Further inquiries can be directed at the corresponding author.

## Footnotes

^1^
An overview of how the autopoietic-enactive approach differs from other integrative approaches – and its respective advantages – is provided by De Haan [25].

^2^
In this paper, we consistently refer to the *autopoietic-enactive approach*. Please note, however, that enactivism forms a rapidly evolving body of thought in which we now see that a distinction is made between *autopoietic, sensorimotor*, and *radical enactivism* [34]. Autopoietic enactivism is the foundational strand and highlights the role of biodynamics in meaning-making. As the application of enactivism to the philosophy of psychiatry is relatively new, we find that most well-known and dominant enactive theories of psychopathology are based on this strand. *Sensorimotor enactivism* focuses on skillful environmental exploration, while *radical enactivism* promotes a fully non-representation view of cognition. Despite their philosophical differences, all three strands are “united by a common commitment to understanding cognition as rooted in our engaged, bodily lives” [34]. Please note that the implications of their philosophical differences between these strands of enactivism for the philosophy of psychiatry have not yet been systematically studied.

^3^
In autopoietic-enactive terms, we can generally describe ‘stable patterns of engagement’ as patterns that persist over time because they successfully support the individual’s ongoing self-regulatory behavior. By ‘authentic patterns of engagement,’ we mean that these behavioral patterns are subjectively significant to the individual, thus self-endorsed rather than imposed from the outside. Finally, ‘health-promoting’ generally means that the pattern of engagement has a beneficial rather than negative effect on the person’s well-being. These concepts should be viewed in light of two considerations. First, they should be viewed as a practical set of *heuristics* for relevant stakeholders to make shared sense of, explore, and substantiate skillful human-world interactions; they are not intended as strictly defined healthcare *criteria*. Second, we are aware that, as such, they *raise* questions rather than answer them. Take, for example, a fixed route to school. As mentioned in the previous section: when does that fixed route to school become *rigid*? How can this be assessed? Aside from various methodological challenges – which are touched upon in the discussion of this paper – there is also a clear normative challenge here: *who* determines when stability turns into rigidity? And is rigidity necessarily bad? Overall, who determines when something is generally considered ‘healthy’ rather than ‘unhealthy’ behavior? These questions are discussed in the context of the ‘demarcation problem’ in the philosophy of psychiatry [53] – questions that we do not intend to answer here but rather draw explicit attention to using the frameworks fifth dimension: aspects of *sociocultural life*.

^4^
In parallel to this paper, we conducted a scoping review to map autism healthcare practices that are already based on autopoietic-enactivism (*explicitly*) or bear with affinity with it (*implicitly*) [156]).

^5^
Eve is a pseudonym.

^6^
In the context of this study, it is moreover important to mention that an autopoietic-enactive approach does not provide a normative or ethical framework. Despite its emphasis on situatedness and interactivity, social model or neurodiversity approaches to autism support needs are not *necessarily* implied by it [157, 158]. Key is that, while an autopoietic-enactive emphasizes the integration of various dimensions of care, it makes no suggestions on when and where to put the emphasis on which dimension. In other words, the ethical idea that also the environment should adapt to the individual – rather than only the other way around – may accompany an autopoietic-enactive approach but is not entailed by it.
